# A Single Catalyst for Promoting Reverse Processes: Synthesis and Chemical Degradation of Polylactide

**DOI:** 10.1002/cssc.202101518

**Published:** 2021-11-02

**Authors:** Federica Santulli, Marina Lamberti, Mina Mazzeo

**Affiliations:** ^1^ Dipartimento di Chimica e Biologia “Adolfo Zambelli” University of Salerno via Giovanni Paolo II SA 132-84084 Fisciano Italy

**Keywords:** circular economy, depolymerization, polylactide, ring-opening polymerization, zinc

## Abstract

A simple zinc catalyst showing high activity for both the synthesis of polylactide, a biodegradable polymer produced from renewable feedstock, and its degradation was described. In the ring‐opening polymerization of lactides, the zinc catalyst showed one of the highest activities reported in the literature for reactions carried out in solution at room temperature. This excellent performance was preserved even when the process was performed under industrial conditions: at high temperature, in the absence of solvent, and by using a low catalyst loading with unpurified monomers. The same complex revealed high efficiency also in depolymerization of polylactide by alcoholysis, a process that occurred efficiently at room temperature and in the absence of solvent, conditions that reduce costs and guarantee low environmental impact.

## Graphical abstract

**Back and forth**: Bioplastics may offer a valid contribution to contrast the environmental impact of the traditional plastic waste, but more virtuous production, disposal, and recycle processes are required. Efficient catalysts, elimination of solvents, purification procedures of monomers, and chemical recycling of the polymeric waste are critical points that must be addressed. In this contribution a zinc complex is described that is able to efficiently promote the synthesis of PLA and, with comparable efficacy, its chemical degradation.

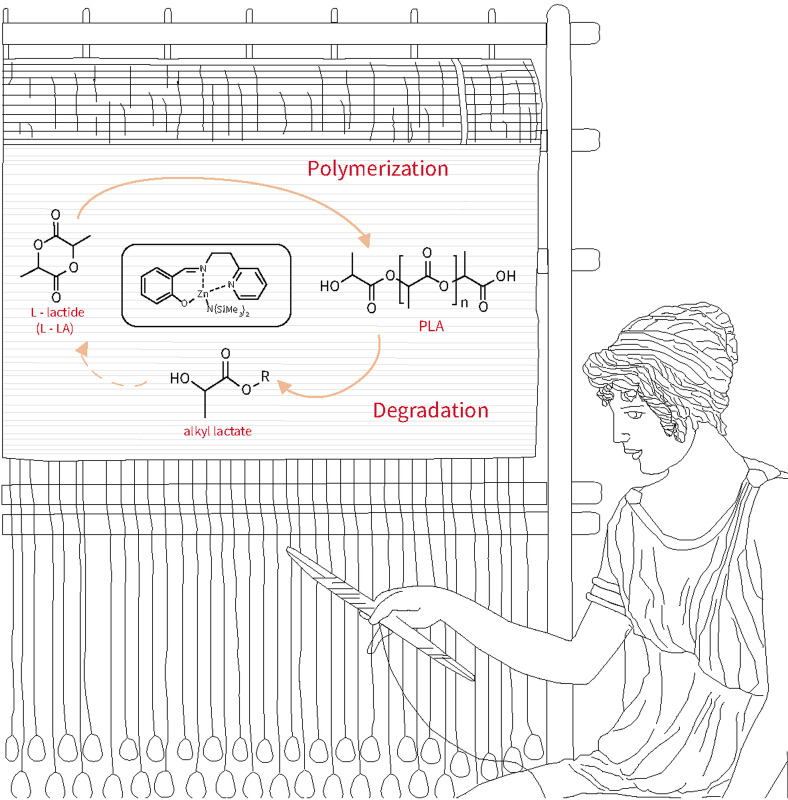

## Introduction

Synthetic oil‐derived polymers are indispensable for modern life and the global economy, but their current linear‐economy model of production as well as their disposal have a dramatic impact on all ecosystems.^[1]^ Every year about 40 % of the global plastic production (more than 400 million metric tons in 2019)^[2]^ is related to single use products and about 50 million tons of plastic waste are disposed into landfills and/or end up in the oceans.^[3]^ The severe consequences of the accumulation of plastic waste in the natural environment stimulated the current scientific research in developing new strategies to either substitute traditional polymers with biodegradable alternatives^[4]^ or effectively recycle postconsumer plastics.^[5]^

Ideally, the implementation of a virtuous model for consumer goods within the circular economy vision requires the convergence of these approaches consisting in the use of bio‐renewable plastics for which procedures of management of their end‐of‐life destiny have been defined ab initio. In this context, catalysis has a leading role.^[6]^

Among the sustainable polymers, polylactide (PLA) is the most commercially promising material because it combines good mechanical properties, biodegradability, and biocompatibility. Moreover, PLA is derived from annually renewable resources, and its life cycle assessment (LCA) suggests a reduction of up to 40 % in greenhouse gas emissions and 25 % in non‐renewable energy use compared to traditional polyolefins.

Although these characteristics define a green profile for PLA, its current production and waste management strategies still show criticisms in terms of sustainability.

At present, the catalyst industrially used to produce PLA via ring‐opening polymerization (ROP) of lactide is tin(II) bis(2‐ethylhexanoate) [Sn(Oct)_2_], which is classified as a toxic compound.

Recently, more benign zinc^[7]^ and magnesium^[8]^ complexes have been introduced as alternative catalysts. They offer several advantages such as high activities and control^[9]^ over the polymerization process and, at the same time, guarantee low costs and no toxicity. Although some complexes reported in the literature were revealed to be extremely active catalysts for ROP of lactide,^[10]^ they often suffer of a certain thermal instability and are unable to preserve their activity under industrially relevant conditions for large‐scale applications.[^10b^, ^11^] Only rare examples of zinc catalysts preserve their high activity under industrially relevant conditions.^[12]^

Ideally, the development of a sustainable process for a large‐scale use of bioplastics requires not only the implementation of an efficient and environmental benign synthetic procedure but also a fruitful reconversion of products and materials at end‐of‐life. Currently, the recycling of PLA is not effectively planned, and its waste management strategy is still aligned with a linear economic model.^[8]^

In this context, the chemical recycling of PLA by conversion into useful chemicals by depolymerization or degradation reactions is a particularly attractive route.^[13]^ For this purpose, different methods have been reported in the literature, including hydrolytic^[14]^ and thermal degradation,^[15]^ as well as enzymatic processes.^[16]^

Among these, the alcoholysis of PLA^[17]^ to obtain alkyl lactates is a very promising route since these products can be used as green substitutes of oil‐derived solvents^[18]^ or, alternatively, may represent fruitful chemical platforms to produce lactic acid, which can be reintroduced in the production cycle of PLA or used for conversion into other chemicals. Thus, this approach would offer the additional advantage of reducing the production cost of PLA and its impact on the food supply chain. Zinc catalysts^[19]^ and organocatalysts^[20]^ have been successfully applied in this field.

Recently, Jones et al. demonstrated that homoleptic Zn^II^‐complexes are highly active catalysts for PLA synthesis and methanolysis, under mild reaction conditions.^[21]^

Herein we report the synthesis and characterization of a heteroleptic pyridyl imino‐phenolate zinc complex and its use as catalyst for the ROP of lactide both in solution and under industrially conditions. The behavior of this catalyst in the degradation of PLA by alcoholysis is also described.

## Results and Discussion

**Figure 1:**
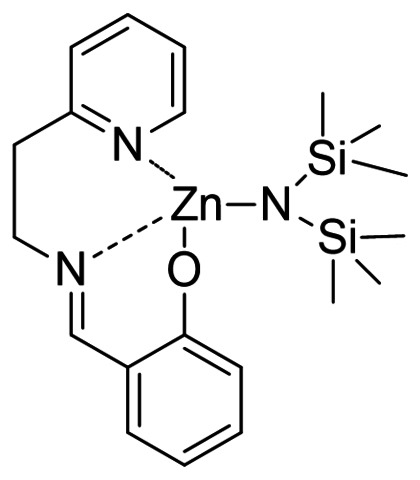
Structure of the catalyst used in this work.

The Zn^II^ complex **1** was obtained by direct reaction between Zn[N(SiMe_3_)_2_]_2_ and the tridentate Schiff base‐like ligand^[22]^ in toluene or THF (Figure 1).

The complex was characterized by mono‐ and bi‐dimensional NMR spectroscopy and elemental analysis (Figures S2–S11).

The ^1^H and ^13^C NMR spectra, recorded both in C_6_D_6_ and CD_2_Cl_2_, showed sharp and well resolved resonances for the pyridyl imino‐phenolate ligand and the bis‐(trimethylsilyl)amide group of the zinc complex, which were consistent with a heteroleptic structure in solution for which no change to the zinc coordination on the NMR timescale was observed. Reliable evidence of coordination of the pyridine moiety to the metal centre was shown by the low‐field ^1^H NMR shift of proton in the *ortho* position^[23]^ of the pyridyl ring (8.46 ppm) in comparison to the same resonance for the free ligand (8.31 ppm) and by the nuclear Overhauser enhancement spectroscopy (NOESY) measurement that showed a strong correlation between the *ortho* proton of the pyridyl fragment and the protons of the bis‐(trimethylsilyl)amide group (Figure S5).

The nuclearity of the complex was assessed via diffusion‐ordered NMR spectroscopy (DOSY) and the result indicated that the complex has a monomeric structure in solution (Figure S8). Thermogravimetric analysis (TGA) showed high thermal stability of complex **1** for more than 1 h at 150 °C (Figure S12).^[24]^

### Polymerization studies

Initial polymerization tests were performed to study the activity of the zinc complex **1** in the ROP of l‐ and *rac*‐lactide in solution at room temperature. Representative results are summarized in Table 1 and additional details are specified in Table S1.

**Table 1:** Polymerization of lactides promoted by **1**.^[a]^

Run	Monomer	[LA]/[Zn]/[ROH]	*t* [min]	*T* [°C]	Conv. [%]	TOF [h^−1^]	*M*_nGPC_^[b]^ [kDa]	*Ð* ^[b]^	*M*_nth_^[c]^ [kDa]
1	*rac*‐LA	100 : 1 : 0	8	25	61	457	2.7	1.70	8.8
2	*rac*‐LA	100 : 1 : 1	1	25	100	2640^[g]^	13.0	1.07	14.4
3	*rac*‐LA	400 : 1 : 1	1	25	77	28320^[g]^	49.5	1.14	44.4
4	l‐LA	400 : 1 : 1	1	25	70	25920^[g]^	45.1	1.12	40.3
5	l‐LA	400 : 1 : 40	2	25	97	14880^[g]^	1.80	1.10	1.44
6	l‐LA	400 : 1 : 100	2	25	67	8040	0.9	1.10	0.4
7^[d]^	l‐LA	1000 : 1 : 2	2	25	56	16800	37.8	1.30	40.3
8^[e]^	l‐LA	1000 : 1 : 2	2	25	68	20400	31.2	1.37	48.9
9^[f]^	l‐LA	1000 : 1 : 0	4	150	23	3450	46.9; 28.2	bimodal	33.2
10^[f]^	l‐LA	1000 : 1 : 10	1	150	100	60000	9.7	1.38	14.4
11^[f]^	l‐LA	1000 : 1 : 50	0.5	150	100	120000	3.1	1.91	2.9
12^[f]^	l‐LA	5000 : 1 : 50	5	150	82	49200	8.9	1.44	11.5

[a] All reactions were carried out by using 10 μmol of Zn catalyst at 25 °C in CH_2_Cl_2_ (2 mL), ROH was *i*PrOH for runs at 25 °C and BnOH for runs at 150 °C. [b] Experimental *M*_n_ (corrected using factor of 0.58) and *Ð* values were determined by gel‐permeation chromatography (GPC) in THF using polystyrene standards. [c] Calculated *M*_nth_=144.14×([LA]/[ROH])×conv. of LA. [d] LA purified by a single crystallization. [e] Unpurified LA. [f] Technical‐grade purity LA, solvent free. [g] TOF calculated after 0.5 min, conversions reported in Table S1.

An initial polymerization experiment of *rac*‐LA was performed at room temperature in methylene chloride. Under these conditions, the zinc catalyst showed a good activity and a turnover frequency (TOF) of 457 h^−1^ (run 1, Table 1). By adding an equivalent of alcohol, the activity increased considerably reaching a TOF up to 28320 h^−1^ (runs 2 and 3, Table 1) both for *rac*‐ and l‐LA (run 4, Table 1). The high activity was preserved also in the presence of a coordinating solvent such as THF (TOF=12000 h^−1^, see Table S1).

The activity showed by this catalyst was higher than those obtained with related zinc complexes bearing pyridyl‐amino‐phenolate ligands. This could be a consequence of the higher donating ability of the sp^2^ imine nitrogen in comparison to sp^3^ amine nitrogen or due to steric effects, such as the different substitutions on the phenoxy ring or the lack of substituent on the imino‐nitrogen and less steric bulkiness of the pendant pyridyl.^[25]^

Increasing the alcohol/ complex ratio up to 100 equivalents (runs 5 and 6, Table 1), in the so called “immortal” conditions, the high activity of catalyst was preserved. Matrix‐Assisted Laser Desorption Ionization Time of Flight (MALDI‐ToF; Figures S13 and S14) analysis of samples revealed single series of peaks with a constant peak spacing of 144 Da indicating the complete absence of transesterification side reactions. The chain end groups were those expected: BnO or *i*PrO and OH groups as observed also by ^1^H NMR spectroscopy (Figure S15).

To align with industrial practices and demonstrate the robust nature of the catalyst, subsequent polymerization experiments were performed by using poorly purified or unpurified monomer (runs 7 and 8 of Table1), and we were delighted to observe no decline of the catalyst performance (TOF=20400 h^−1^) and only a minimal impact on the dispersity of the molecular masses of the obtained polymers.

Subsequently, additional polymerization tests were carried out with l‐LA of technical grade purity at 150 °C with a monomer/metal ratio up to 5000 : 1 (runs 9–12, Table 1) obtaining very high activities in all cases and TOFs up to 120000.

In the absence of alcohol, a bimodal distribution of the molecular masses was observed (run 9, Table 1), while all polymers obtained with **1**/ROH showed monomodal distributions with narrow dispersities and molecular masses values coherent with the theoretical ones thus suggesting a good control of the polymerization process even under challenging conditions (see Figure S16).

Regarding microstructures of the obtained polymers, atactic PLAs were produced by *rac*‐lactide while perfectly isotactic PLAs were obtained by l‐LA even carrying out the polymerizations at high temperatures indicating the absence of epimerization phenomena (see Figures S17 and S18).

Coherently, differential scanning calorimetry (DSC) analysis of the sample produced showed melting temperatures of 164.1 °C, a value indicative of a PlLA with high crystallinity (see Figure S19).

### Depolymerization studies

The chemical recycling of PLA via transesterification by methanol or ethanol allows the formation of valuable low‐molecular‐weight compounds, such as methyl and ethyl lactate, respectively. These can be used as biodegradable “green” solvents with low toxicity and find large application in different fields such as pharmaceutical and food industry.^[26]^

Additionally, alkyl lactates can be reconverted into lactic acid, offering the opportunity to implement a circular economy model with beneficial effects on the costs of the PLA production and reducing the risk of impact on the food supply chain.

Initially, we investigated the degradation by methanolysis of several samples of PLA, different for stereoregularity [isotactic poly(l‐LA) and atactic poly(rac‐lactide)] or for molecular masses. Some commercial products were also selected. The details of the PLA samples tested are summarized in Table S2 in the Supporting Information.

**Figure 2:**
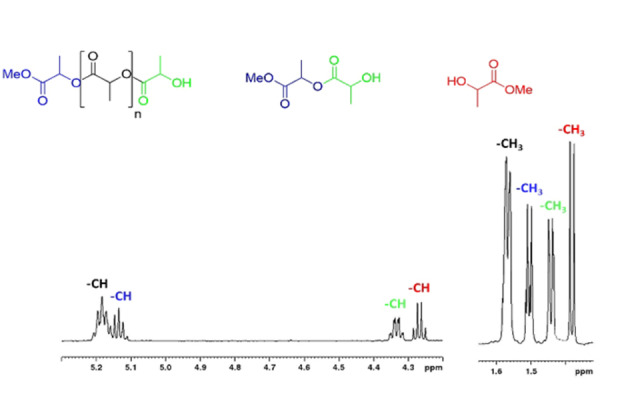
^1^H NMR spectrum (CD_2_Cl_2_, 25 °C) of a degradation reaction of PLLA

All depolymerization reactions were performed at room temperature with 0.6 mol % catalyst with respect to the repeating units of polymer (Tables 2 and S3) and by using unpurified solvent and alcohol. The monitoring of the degradation reaction was performed by regular sampling and ^1^H NMR spectroscopy (Figure 2) of the reaction. In the spectral region between 4.00 and 5.20 ppm, four different signals were evident, attributable to methine protons of the chain end units of PLA oligomers, of the internal repeating units and of methyl lactyl‐lactate. Conversion of internal methine units (*X*_int_), methyl lactate selectivity (*S*_Me‐LA_), and methyl lactate yield (*Y*_Me‐LA_) were chosen as representative parameters for a comparative analysis with the studies reported by Sobota and co‐workers^[27]^ and Wood and co‐workers.^[28]^

**Table 2:** Methanolysis of PLAs performed in THF solution.^[a]^

Run	Sample	*M*_n_^[b]^ [kDa]	*t* [h]	*X*_Int_^[c]^ [%]	*S*_Me‐La_^[c]^ [%]	*Y*_Me‐La_^[c]^ [%]
1	PlLA	30	1	100	61	61
2	PlLA	48	1	100	74	74
3	PlLA	92	1	100	77	77
4	PlLA	14	1	97	95	92
5	P(*rac*‐LA)	13	1	44	35	16
6	P(*rac*‐LA)	44	1 2	75 88	69 89	52 78
7^[d]^	PlLA	30	2 4.5	89 97	38 61	34 60
8^[d]^	PLA cup	58	2 4.5	100 100	47 100	47 100
9^[d]^	PLA filament	20	2 4.5	84 100	26 62	22 62

[a] All reactions were carried out by using 5 μmol of Zn catalyst (0.6 mol % relative to ester linkages) in 1.8 mL of THF, with 0.2 mL of MeOH (6 times relative to ester linkages). [b] *M*_n_=experimental molecular weight (GPC). [c] Determinated by ^1^H NMR spectroscopy. [d] Reaction performed in air.

The zinc complex **1** revealed high activity in the degradation of isotactic PlLA samples with molecular masses ranging from 14.0 to 90.0 KDa, and in all cases the quantitative degradation of polymers was achieved after 1 h at room temperature (runs 1–4, Table 2).^[29]^

**Figure 3:**
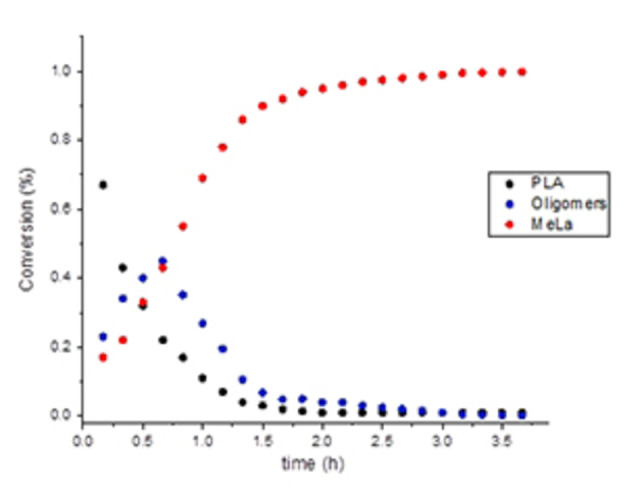
Kinetic profile for degradation of a PLLA sample. [PLA]_0_=0.44 m; [polyester linkages]_0_/[Zn]_0_=60; THF‐d8 (0.45 mL) as solvent; MeOH 0.05 mL.

**Scheme 1:**
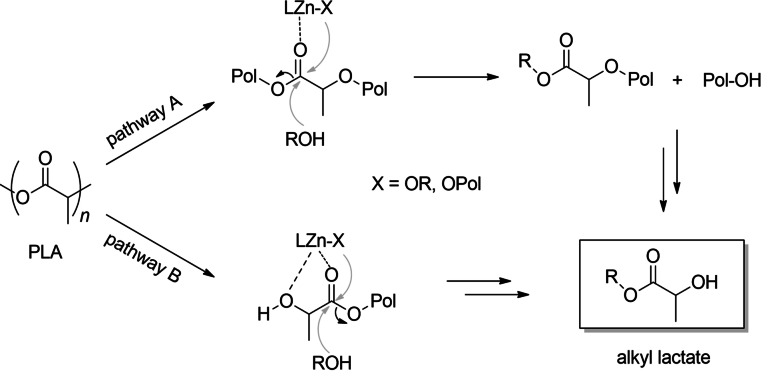
Alcoholysis of PLA via random scission of the chains (pathway A) or by chain‐end cutting (pathway B).

The NMR monitoring (Figure 3) showed that degradation process occurred via a two‐step process (pathway A of Scheme 1) in which a random scission of the polymer chains in the initial stages led to the formation of oligomeric species that are progressively converted into methyl lactate.

For reactions performed in THF solution, the degradation rate was revealed to be not depending on the molecular weight (see runs 1–4, Table 2), coherently with a mechanism of random scission of the polymeric chain (pathway A of Scheme 1). In this mechanism the zinc acts as a Lewis acid and activates a carbonyl group of the chain for a nucleophilic attack by the alkoxide labile group or an external alcohol. Surprisingly, the degradation rate was influenced by the tacticity of the polymer (see run 4 vs. 5 and run 2 vs. 6, Table 2), and it was faster for the isotactic than for atactic PLAs. Probably, the formation of different diastereoisomer zinc species during the degradation reactions may be responsible for the observed results.

**Figure 4:**
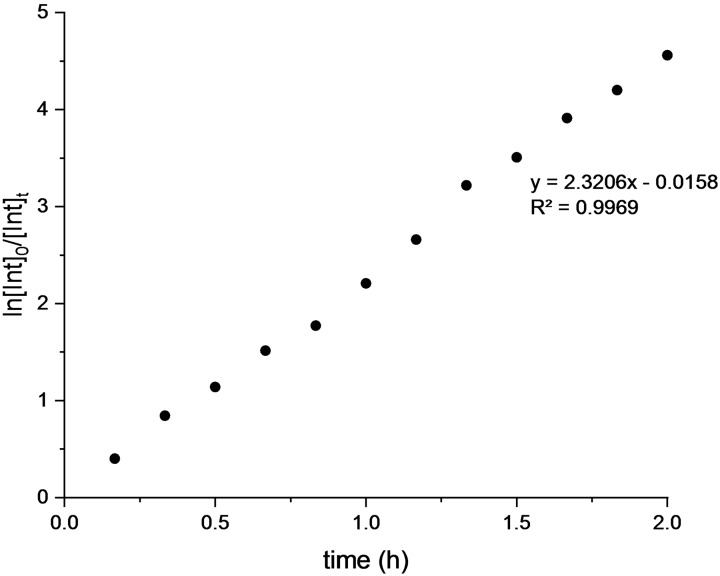
Pseudo‐first‐order kinetic plot for degradation of polylactide. [PLA]_0_=0.44 m; [polyester linkages]_0_/[Zn]_0_=60; THF‐d8 (0.45 mL) as solvent; MeOH 0.05 mL. Pseudo‐first‐order rate is 2.32 h^−1^ at 25 °C (*R* ^2^=0.9969).

The NMR analysis of PLLA degradation performed in THF‐d8 showed a linear relationship of ln([PLLA]_0_/[ PLLA]_*t*_) against time indicative of a first‐order kinetics for the process with a *k*_app_ value of 2.3 h^−1^ at 25 °C (Figure 4). A similar value of *k*_app_ was obtained for degradation of atactic PLA (Figures S21 and S22).

The good performances of complex **1** were preserved when the reactions were performed in the presence of air; in fact, only a little decrease of the degradation rate was observed (runs 1 vs. 7, Table 2).

To demonstrate the applicability of this complex in recycling processes of post‐consumer PLA samples, degradation experiments of an end‐of‐life PLA plastic cup and a PLA filament for 3D printing were performed in air under the conditions of run 7 of Table 2. The PLA plastic cup was rapidly converted to methyl lactate after about 4 h at room temperature (run 8, Table 2). Lower activity was observed with 3D printing filament, reasonably as consequence of the presence of unknown additives in the sample (run 9, Table 2).

Subsequently, alcoholysis experiments were performed without the use of additional solvents (Table 3).

**Table 3:** Alcoholysis of polylactides under solvent‐free conditions.^[a]^

Run	Sample	ROH	*M*_n_^[b]^ [kDa]	*t* [h]	*X*_Int_^[c]^ [%]	*S*_Me‐La_^[c]^ [%]	*Y*_Me‐La_^[c]^ [%]
1	P(*rac*‐LA)	MeOH	38	1	100	100	100
2	PlLA	MeOH	30	3 8	51 100	100 100	51 100
3	PlLA	MeOH	48	3 8	36 75	100 100	36 75
4	PlLA	MeOH	92	3 8	25 79	100 100	25 79
5	PlLA	MeOH	160	24	100	100	100
6	PlLA	EtOH	92	24	100	80	80
7 ^d^	PlLA	EtOH	92	13	100	100	100

[a] All reactions were carried out by using 5 μmol of Zn catalyst (0.6 mol % relative to ester linkages) in 1.8 mL of THF, with 0.2 mL of MeOH (6 times relative to ester linkages) [b] *M*_n_=experimental molecular weight (GPC). [c] Determinated by ^1^H NMR spectroscopy. [d] Reaction performed at reflux temperature.

The degradation in neat alcohol of P(*rac*‐LA) proceeded very quickly: after 1 h the complete conversion into methyl lactate was observed (run 1, Table 3). As expected, for the crystalline PlLA the degradation time was longer because of its scarce permeability to the methanol, and it was significantly dependent on the molecular masses (see runs 2–5, Table 3). However, even a very high molecular weight PlLA (160.0 KDa) was fully degraded at room temperature, after 24 h.

**Figure 5:**
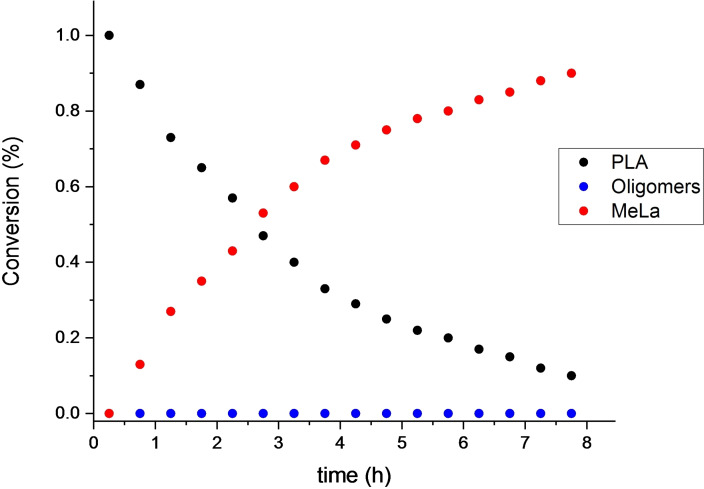
Kinetic profile for degradation of a PlLA sample. [PLA]_0_=0.44 m; [polyester linkages]_0_/[Zn]_0_=60. MeOH 0.50 mL.

**Figure 6:**
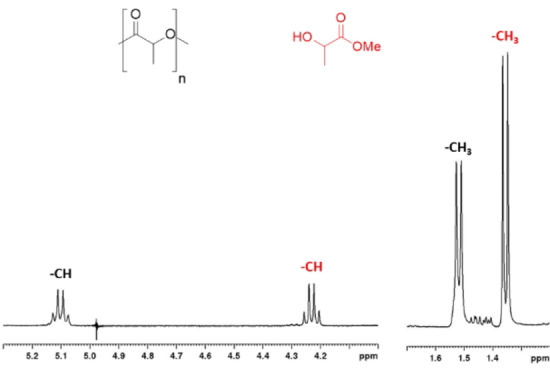
^1^H NMR spectrum in CDCl_3_ of a degradation reaction of PLLA in solvent‐free conditions.

The NMR monitoring of the methanolysis performed in the absence of solvent (Figures 5 and 6) revealed a mechanism in which the degradation occurs via a progressive erosion of the chain ends with the direct formation of methyl lactate (pathway B, Scheme 1).^[30]^ This could be due to the easier accessibility of polymer chain end groups because of hydrogen bond interaction with alcohol.

**Figure 7:**
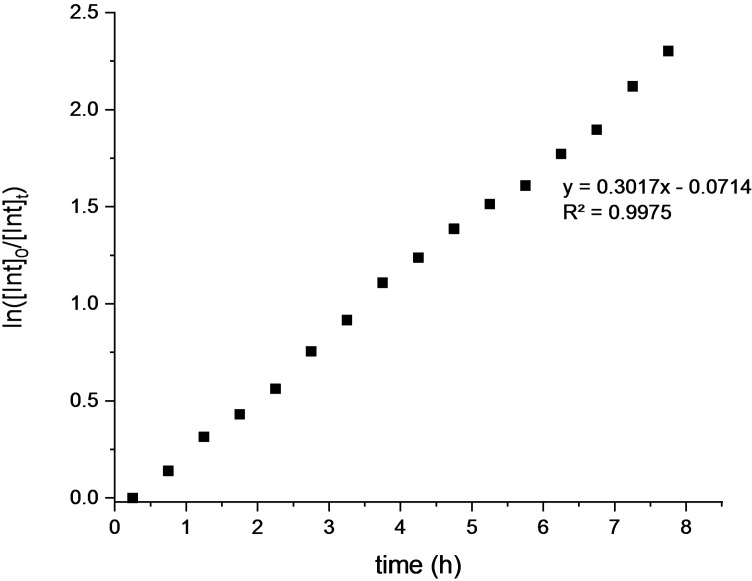
Pseudo‐first‐order kinetic plot for solvent free degradation of polylactide. [PLA]_0_=0.44 m; [polyester linkages]_0_/[Zn]_0_=60; MeOH 0.50 mL. Pseudo‐first‐order rate is 0.30 h^−1^ at 25 °C (*R* ^2^=0.9975).

A pseudofirst‐order kinetic plot described also the solvent‐free degradation of polylactide (Figure 7).

The ethanolysis reaction resulted to be slower than methanolysis; however, after 24 h at room temperature the degradation of the polymer was complete with a 80 % yield of ethyl lactate, while at reflux temperature, only 13 h were necessary to quantitatively convert the polymer to ethyl lactate (runs 6 and 7, Table 3).

## Conclusion

A very simple Schiff base zinc complex has been prepared and used as catalyst for the ring‐opening polymerization (ROP) of lactides. Excellent activity and efficient control were observed in reactions performed in solution at room temperature. These successful abilities were preserved even under industrially relevant conditions such as high temperature, low catalyst loading, and with unpurified monomers. The activities obtained classify this system among the most efficient catalysts reported in the literature and comparable to the industrial catalyst Sn(Ot)_2_.

The same complex was successfully employed for the degradation of high‐molecular‐weight and crystalline poly(l‐lactide) (PlLA) and of post‐consumer PLA products.

The full sustainability of both processes was guaranteed by the non‐toxic nature of zinc and by environmentally friendly reaction conditions: absence of solvent and low catalyst loading. Further studies are ongoing on this class of catalytic systems, whose versatility met the demands of a circular economy model for PLA.

## Experimental Section

### Synthesis and characterization of complex 1

To a stirred solution containing zinc bis[bis(trimethylsilyl)amide] (773 mg, 2.0 mmol) in dry benzene (4.0 mL) was added dropwise a solution of the ligand precursor (453 mg, 2.0 mmol) in dry benzene (2.0 mL). The solution was stirred for 3 h at room temperature. The solvent was removed under vacuum, forming a red solid. Yield: 80 %. The formation of the desired species was confirmed by NMR spectroscopy and elemental analysis. Elemental analysis calculated for: C_20_H_31_N_3_OSi_2_Zn (%): C, 53.26; H, 6.93; N, 9.32; Found: C, 52.98; H, 7.02; N, 9.47.

### General procedure for the polymerization of lactide at room temperature

The polymerization was carried out under inert conditions. In a Braun Labmaster glovebox, a magnetically stirred reactor vessel (10 mL) was charged with lactide. The metal complex and *i*PrOH (0.1 m solution of *i*PrOH in dichloromethane) were added in a 4 mL vial and mixture was stirred for 5 minutes; then dichloromethane was added, and the monomer was transferred in. The reaction mixture was stirred at room temperature. During the reaction, small aliquots of the reaction mixture were sampled, dissolved in CDCl_3_, and analyzed by ^1^H NMR spectroscopy. At the end of the polymerization the product was precipitated in hexane, then filtered and dried in a vacuum oven.

### General procedure for the polymerization of lactide at high temperature

The polymerization was carried out under inert conditions. In a Braun Labmaster glovebox, a magnetically stirred reactor flask (50 mL) was charged with lactide. The metal complex and 1 equivalent of BzOH (0.1 m solution of BnOH in toluene) were added in a 2 mL vial and the mixture was stirred for 5 minutes, then the monomer was transferred in, and finally an additional amount of BnOH was added as specified in different runs of Table 1. The reaction mixture was stirred at 150 °C. During the reaction, small aliquots of the reaction mixture were sampled, dissolved in CDCl_3_, and analyzed by ^1^H NMR spectroscopy. At the end of the polymerization the product was precipitated in hexane, then filtered and dried in a vacuum oven.

## Conflict of interest

The authors declare no conflict of interest.

## Supporting information

As a service to our authors and readers, this journal provides supporting information supplied by the authors. Such materials are peer reviewed and may be re‐organized for online delivery, but are not copy‐edited or typeset. Technical support issues arising from supporting information (other than missing files) should be addressed to the authors.

Supporting Information
