# Supplementary material for: A Single Catalyst for Promoting Reverse Processes: Synthesis and Chemical Degradation of Polylactide
Source: ChemSusChem. 2021 Nov 2;14(24):5470–5. doi: 10.1002/cssc.202101518 (PMC9298063; doi:10.1002/cssc.202101518)
Supplement: Supplementary file 1 — Supporting Information [file CSSC-14-5470-s001.pdf]

ChemSusChem

Supporting Information

A Single Catalyst for Promoting Reverse Processes: Synthesis and Chemical Degradation of Polylactide

Federica Santulli, Marina Lamberti, and Mina Mazzeo*© 2021 The Authors. ChemSusChem published by Wiley-VCH GmbH. This is an open access article under the terms of the Creative Commons Attribution License, which permits use, distribution and reproduction in any medium, provided the original work is properly cited.

Experimental section

Figure S1. ^{1}H NMR spectrum (400 MHz, C_6D_6, 298 K) of ligand *L1* 2

Figure S2. ^{1}H NMR spectrum (400 MHz, C_6D_6, 298 K) of complex **1**. 3

Figure S3. ^{13}C NMR spectrum (100 MHz, C_6D_6, 298 K) of complex **1**. 4

Figure S4. Aromatic region of COSY NMR spectrum (400 MHz, C_6D_6, 298 K) of complex **1**. 4

Figure S5. NOESY NMR spectrum (600 MHz, C6D6, 298 K) of complex 1. 5

Figure S6. Aromatic region of HSQC NMR spectrum (600 MHz, C_6D_6, 298 K) of complex **1**. 5

Figure S7. Aliphatic region of HSQC NMR spectrum (600 MHz, C_6D_6, 298 K) of complex **1**. 6

Figure S8. DOSY NMR spectrum (400 MHz, C_6D_6, 298 K) of complex **1** (D = 7.73 10^{-10} m^2/s) in the presence of tetrakis(trimethylsilyl)silane TMSS as standard (D = 9.59 10^{-10} m^2/s). 6

Figure S9. ^{1}H NMR spectrum (400 MHz, CD_2Cl_2, 298 K) of complex **1**. 7

Figure S10. ^{13}C NMR spectrum (400 MHz, CD_2Cl_2, 298 K) of complex **1**. 8

Figure S11. Aromatic region of COSY NMR spectrum (400 MHz, CD_2Cl_2, 298 K) of complex **1**. 8

Figure S12. TGA measurement of the complex **1** at 150°C. 9

Figure S13. MALDI-TOF of P(Lrac-LA) sample run 2 Table 1. 11

Figure S14. MALDI-TOF of P(L-LA) sample run 11 Table 1 10

Figure S15. Methine region of the homonuclear decoupled ^{1}H NMR spectrum ($CDCl_3$, RT, 400MHz) of a P(arc-LA) sample. 13

Figure S16. GPC analysis of sample 12 Table 1. 11

Figure S17. Methine region of the homonuclear decoupled ^{1}H NMR spectrum ($CDCl_3$, RT, 400MHz) of a P(L-LA) sample run 12 Table 1. 12

Figure S18. Methine region of the homonuclear decoupled ^{1}H NMR spectrum ($CDCl_3$, RT, 400MHz) of a P(D,L-LA) sample run 2 Table 1. 13

Figure S19. DSC measurement of a P(L-LA) sample run 10 Table 1. 14

Table S1. Polymerization of lactides promoted by 1.[a] 14

Table S2. Number average molecular weight (Mn), dispersity (Đ) and Tf of PLA samples used for depolymerization reactions. 15

Figure S20. ^{1}H NMR spectrum (600 MHz, CD_2Cl_2, 298 K) depolymerized PLLA. 16

Figure S21. Kinetic profile for degradation of a poly(D,L) lactide sample ([PLA]0 = 0.44 M; [polyester linkages]0/[Zn]0 = 60; CH_2Cl_2 (0.45 mL) as solvent; MeOH 0.05mL). 16

Figure S22. Pseudofirst-order kinetic plot for degradation of poly(rac) lactide ([PLA]0 = 0.44 M; [polyester linkages]0/[Zn]0 = 60; CH_2Cl_2 (0.45 mL) as solvent; MeOH 0.05mL). Pseudofirst-order rate is = 1.50 h^{-1} at 25°C (R^2 = 0.9962). 17

Figure S23. ^{1}H NMR spectrum (400 MHz, $CDCl_3$, 298 K) of methyl-lactide (Me-La). 18

Figure S24. ^{1}H NMR spectrum (400 MHz, C_6D_6, 298 K) of ethyl-lactide (Et-La). 19

Table S3. Methanolysis of PLAs performed in THF solution.[a] 20

Synthesis and characterization of the ligand L1

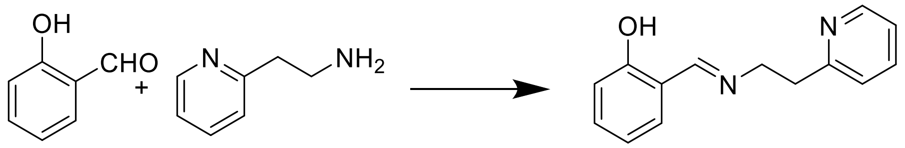

Scheme1. Synthesis of ligand L1.

The ligand was synthetized by condensation of the 2-(2-pyridyl) ethylamine (660 mg, 5.40 mmol) with the salicyl aldehyde (660 mg, 5.40 mmol). The reaction was performed in reflux of ethanol (40 mL) overnight. The solvent was removed under vacuum, forming an orange oil in high yields (81%).

The success of performed reaction was confirmed by ^{1}H NMR experiment, which spectrum is illustrated in the following figure:

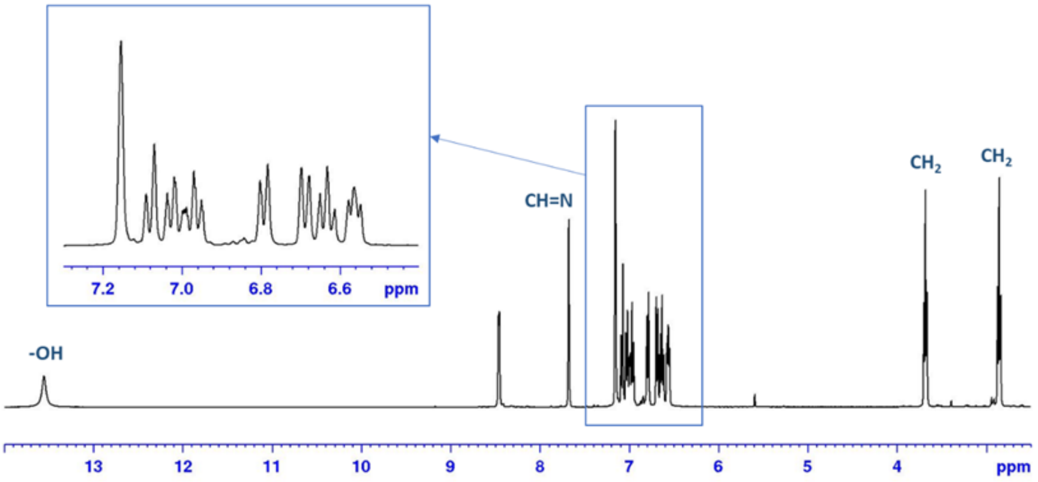

Figure S1. ^{1}H NMR spectrum (400 MHz, C_6D_6, 298 K) of ligand *L1*.

^{1}H NMR (400 MHz, C_6D_6, 298 K): δ 13.6 (s, 1H, -OH), 8.46 (d, J = 4.1Hz, 1H, Py-H), 7.67 (s, 1H, CH=N), 7.08 (d, J = 8.2Hz, 1H, Ph-H), 7.02 (dt, J_1 = 7.2Hz, J_2 = 1.5Hz, 1H, Ph-H), 6.96 (dt, J_1 = 7.5Hz, J_2 = 1.7Hz, 1H, Py-H), 6.79 (d, J = 7.6Hz, 1H, Ph-H), 6.69 (d, J = 7.6Hz, 1H, Py-H), 6.63 (dt, J_1 = 7.2Hz, J_2 = 1.1 Hz, 1H, Ph-H), 6.56 (d, J = 7.2Hz, 1H, Py-H), 3.68 (t, J = 6.9Hz, 2H, CH_2), 2.86 (t, J = 6.9Hz, 2H, CH_2).

Synthesis and characterization of the complex 1

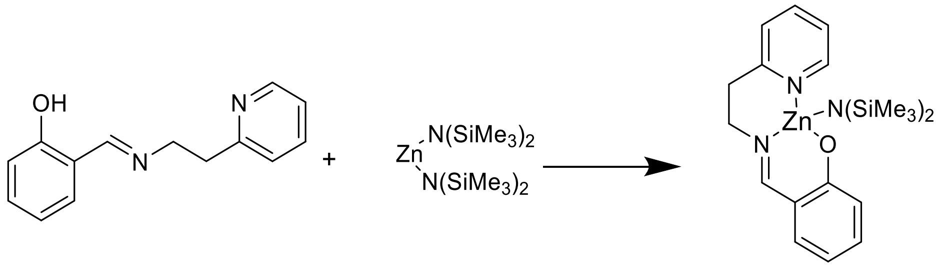

Scheme2. Synthesis of complex **1**.

To a stirred solution containing Zinc bis[bis(trimethylsilyl)amide] (773 mg, 2.0 mmol) in dry benzene (4.0mL) was added dropwise a solution of the ligand precursor L1 (453 mg, 2.0 mmol) in dry benzene (2.0mL). The solution was stirred for 3h at room temperature. The solvent was removed under vacuum, forming a red solid. Yield: 80%. The formation of the desired species was confirmed by NMR and elemental analysis. Elemental Analysis calculated for: C20H31N3OSi2Zn (%): C, 53.26; H, 6.93; N, 9.32; Found: C, 52.98; H, 7.02; N, 9.47.

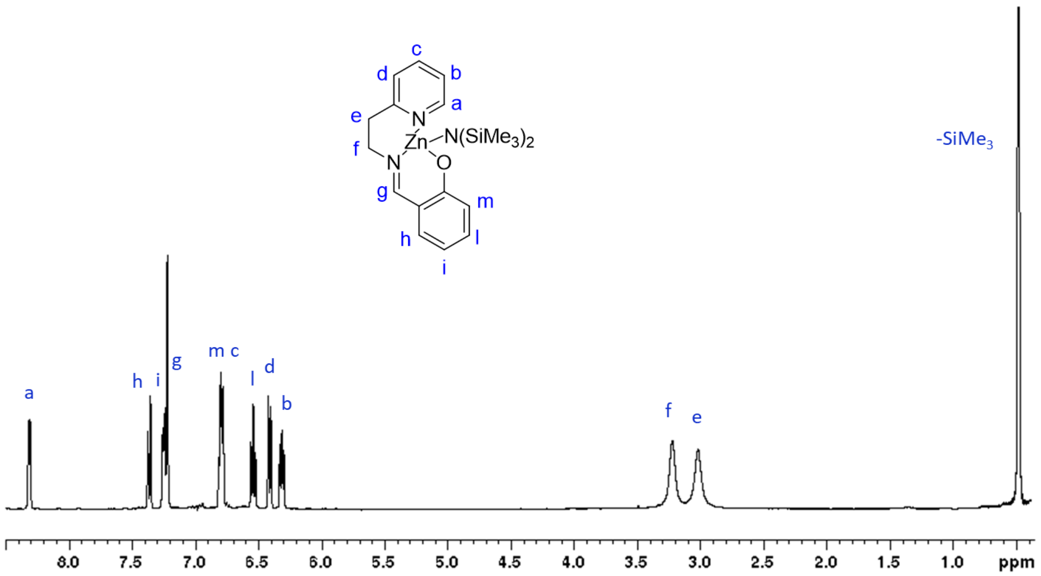

Figure S2. ^1H NMR spectrum (400 MHz, C_6D_6, 298 K) of complex **1**.

^1H NMR (400 MHz, C_6D_6, 298 K): δ 8.31 (d, J = 5.2Hz, 1H, Ha), 7.36 (d, J = 8.6Hz, 1H, Hh), 7.25 (m, 1H, Hi), 7.22 (s, 1H, Hg), 6.80 (m, 2H, Hm-Hc), 6.53 (t, J = 7.8Hz, 1H, Hl), 6.40 (d, J = 7.8Hz, 1H, Hd), 6.31 (t, J = 6.5Hz, 1H, Hb), 3.22 (m, 2H, Hf). 3.01 (m, 2H, He), 0.47 (s, 18H, -$SiMe_3$).

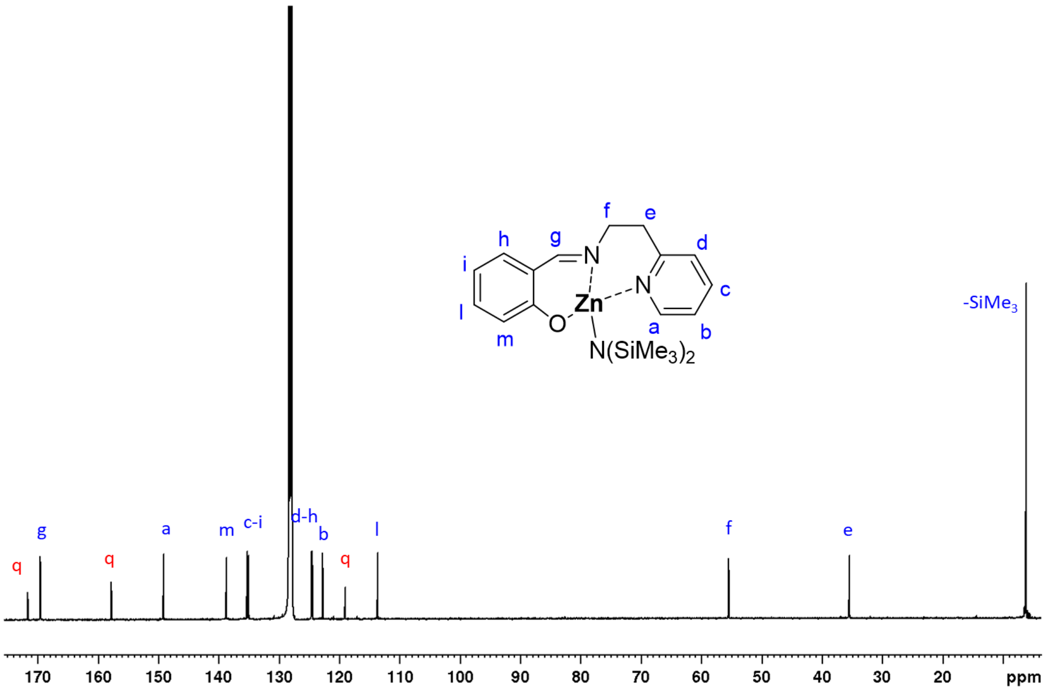

Figure S3. ^{13}C NMR spectrum (100 MHz, C_6D_6, 298 K) of complex **1**.

^{13}C NMR (100 MHz, C_6D_6, 298 K): δ 171.6 (C_q), 169.5 (C_g), 157.8 (C_q), 149.1 (C_a), 138.7 (C_m), 135.2 (C_c), 135.0 (C_i), 124.6 (C_d), 124.4 (C_h), 122.8 (C_b), 119.0 (C_q), 113.6 (C_l), 55.4 (C_f), 35.4 (C_e), 6.12(-$SiMe_3$). Cq means quaternary carbon.

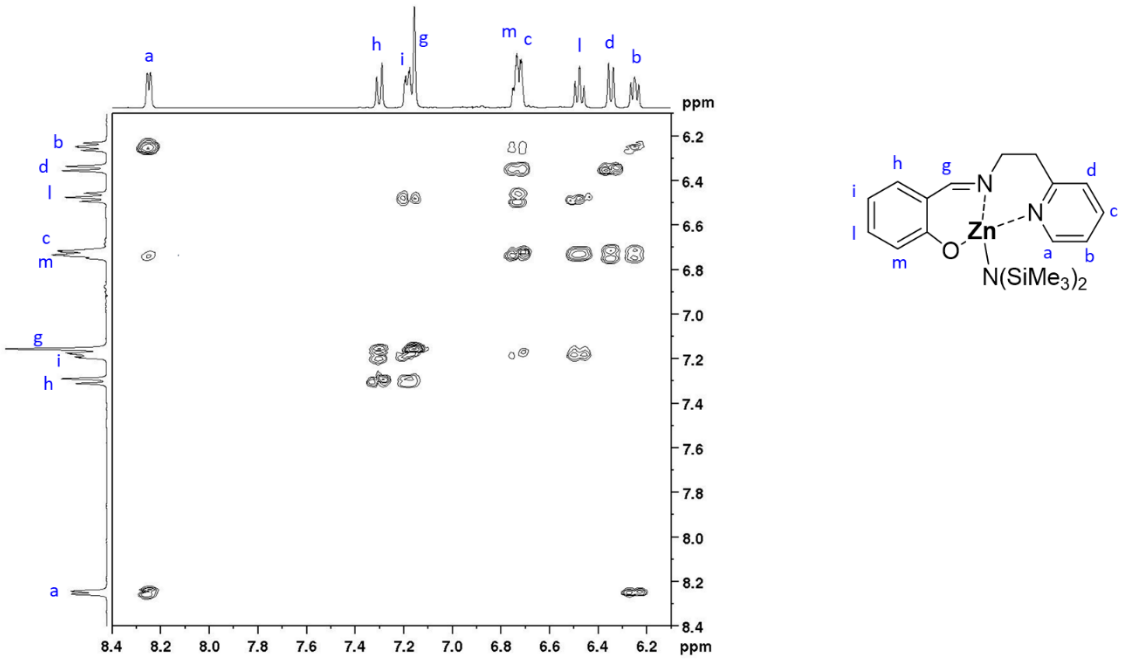

Figure S4. Aromatic region of COSY NMR spectrum (400 MHz, C_6D_6, 298 K) of complex **1**.

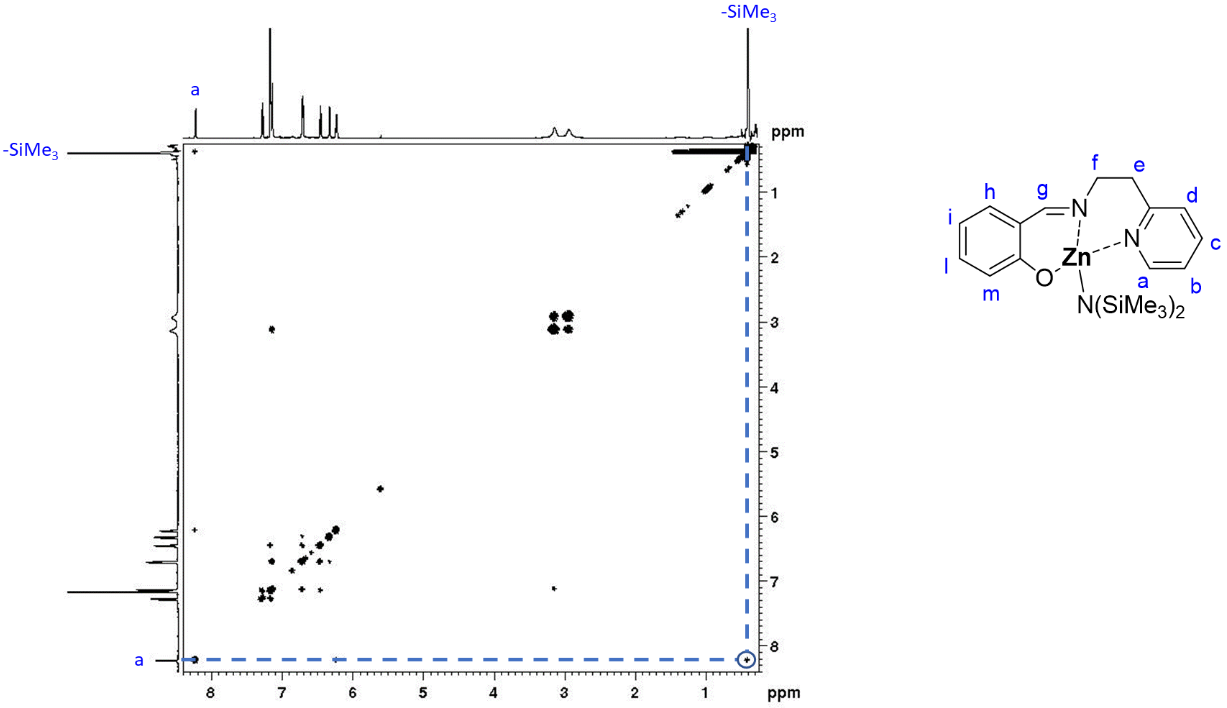

Figure S5. NOESY NMR spectrum (600 MHz, C6D6, 298 K) of complex 1.

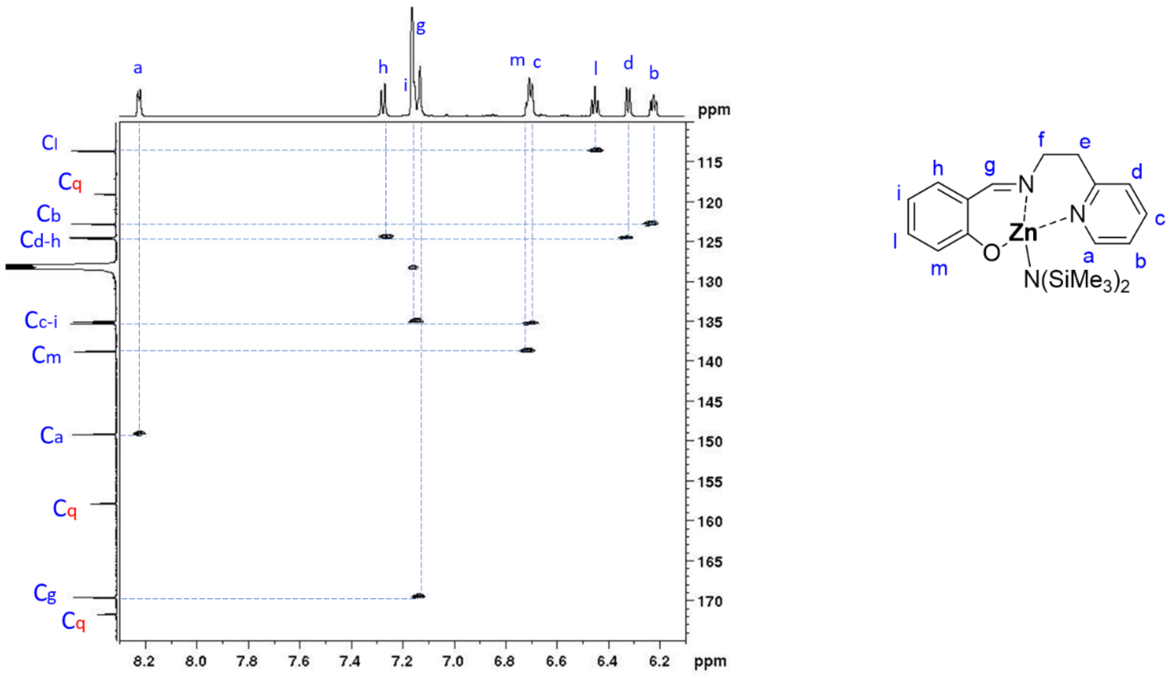

Figure S6. Aromatic region of HSQC NMR spectrum (600 MHz, C_6D_6, 298 K) of complex **1**.

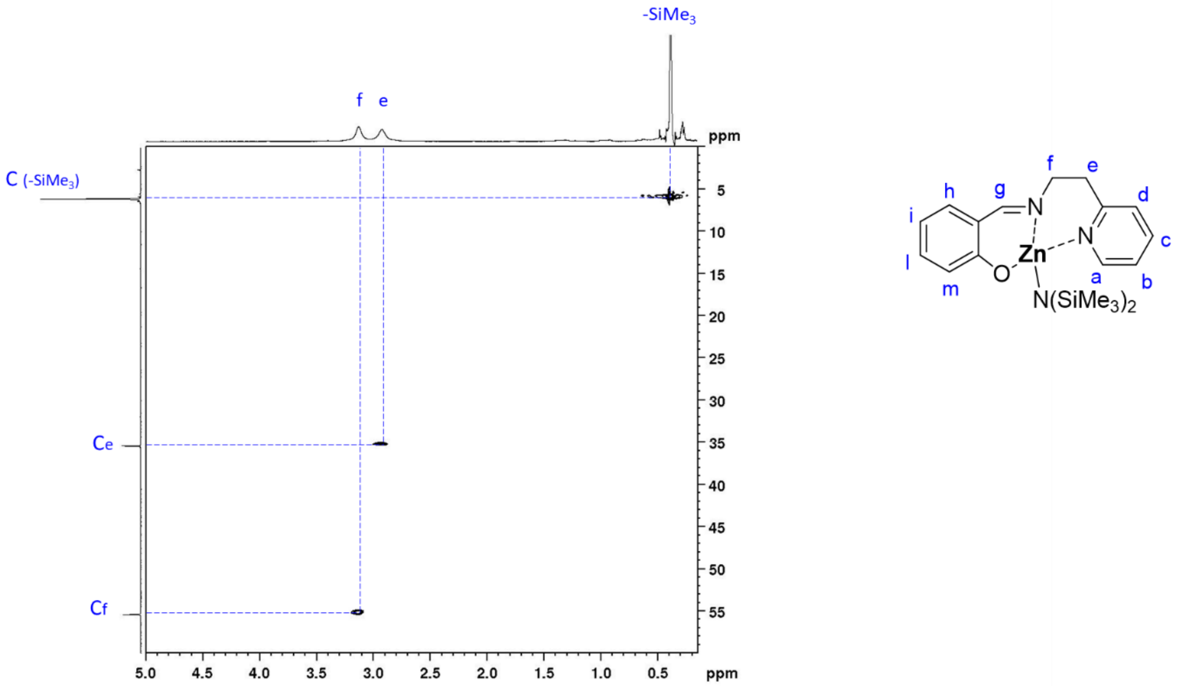

Figure S7. Aliphatic region of HSQC NMR spectrum (600 MHz, C_6D_6, 298 K) of complex **1**.

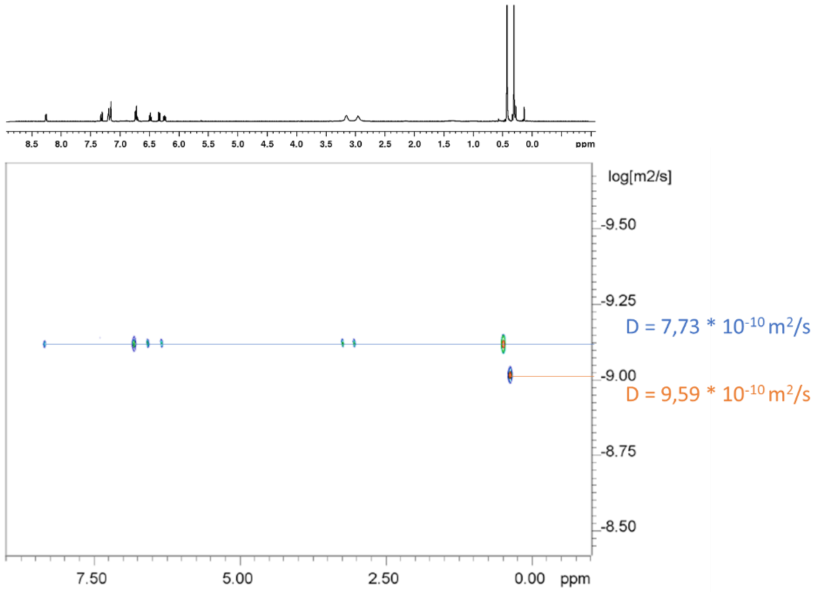

Figure S8. DOSY NMR spectrum (400 MHz, C_6D_6, 298 K) of complex **1** (D = 7.73 10^{-10} m^2/s) in the presence of tetrakis(trimethylsilyl)silane TMSS as standard (D = 9.59 10^{-10} m^2/s).

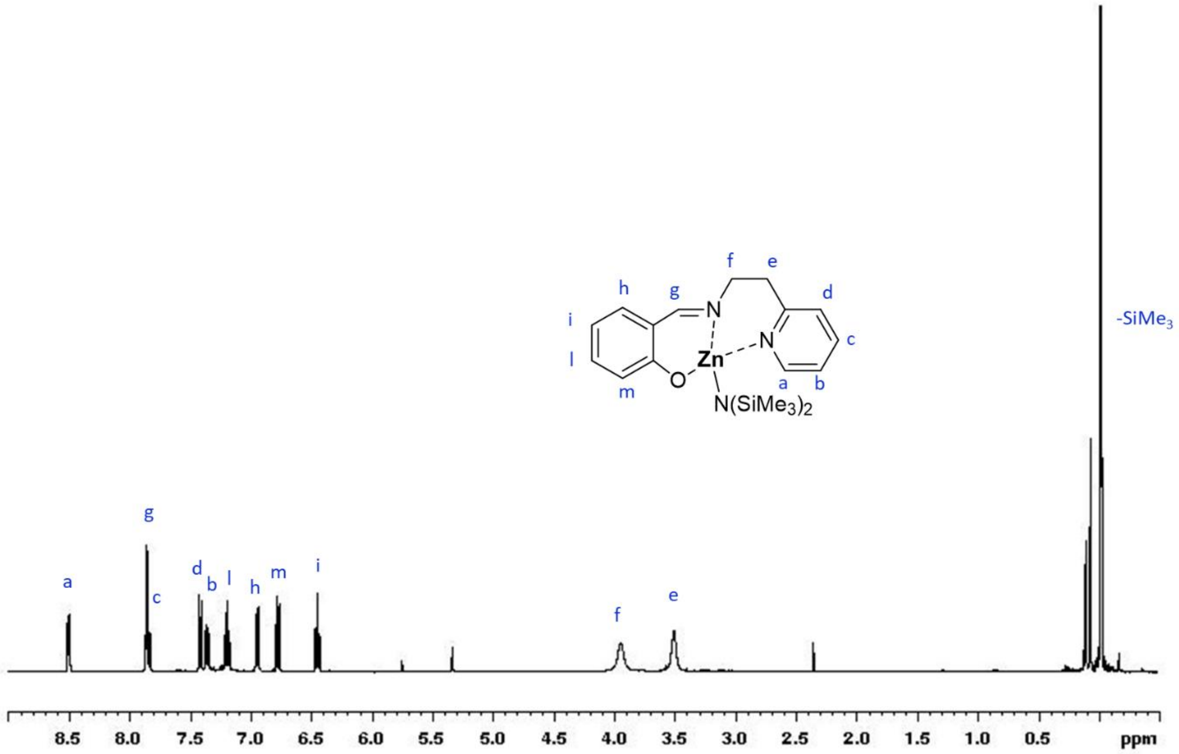

Figure S9. ^{1}H NMR spectrum (400 MHz, CD_2Cl_2, 298 K) of complex **1**.

^{1}H NMR (400 MHz, CD_2Cl_2, 298 K): δ 8.49 (d, J = 5.2Hz, 1H, Ha), 7.84 (m, 2H, Hg-c), 7.41 (d, J = 7.8Hz, 1H, Hd), 7.35 (t, J = 6.5Hz, 1H, Hb), 7.18 (t, J = 8.7Hz, 1H, Hl), 6.94 (dd, J_1 = 7.8Hz, J_2 = 1.7Hz, 1H, Hh), 6.76 (d, J = 8.6Hz, 1H, Hm), 6.44 (t, J = 7.8Hz, 1H, Hi), 3.93 (m, 2H, Hf). 3.49 (m, 2H, He), -0.0336 (s, 18H, -SiMe$_3$).

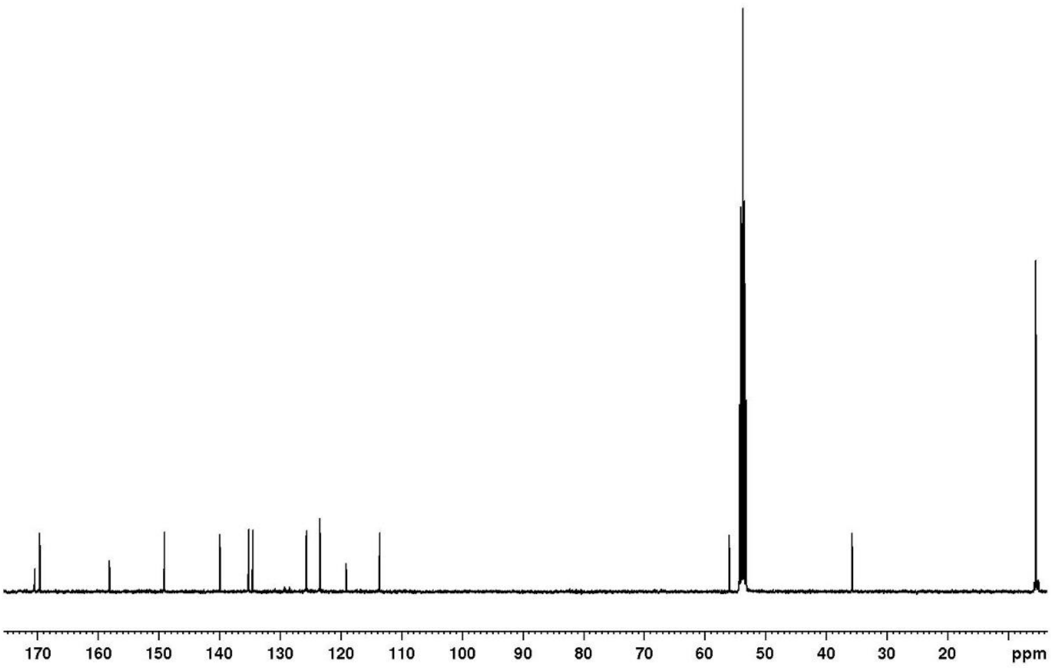

Figure S10. ^{13}C NMR spectrum (400 MHz, CD_2Cl_2, 298 K) of complex **1**.
^{13}C NMR (100 MHz, CD_2Cl_2, 298 K): δ 170.4, 169.6, 158.1, 149.0, 139.9, 135.1, 134.5, 125.6, 123.4, 123.3, 119.1, 113.6, 55.9, 35.7, 5.40.

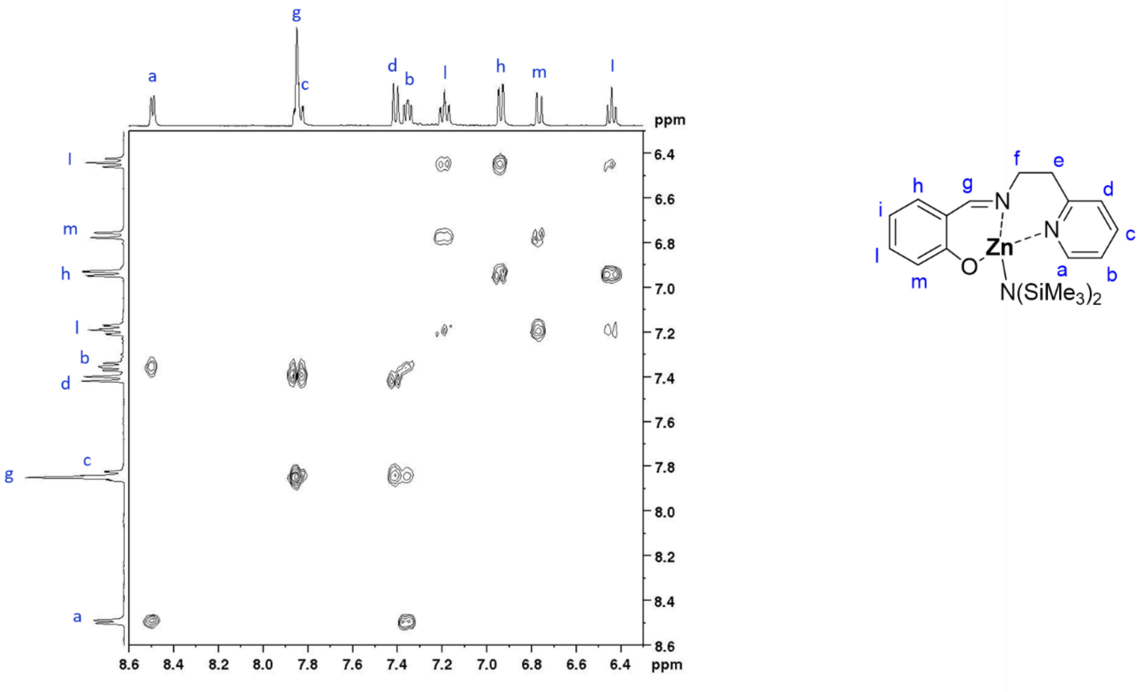

Figure S11. Aromatic region of COSY NMR spectrum (400 MHz, CD_2Cl_2, 298 K) of complex **1**.

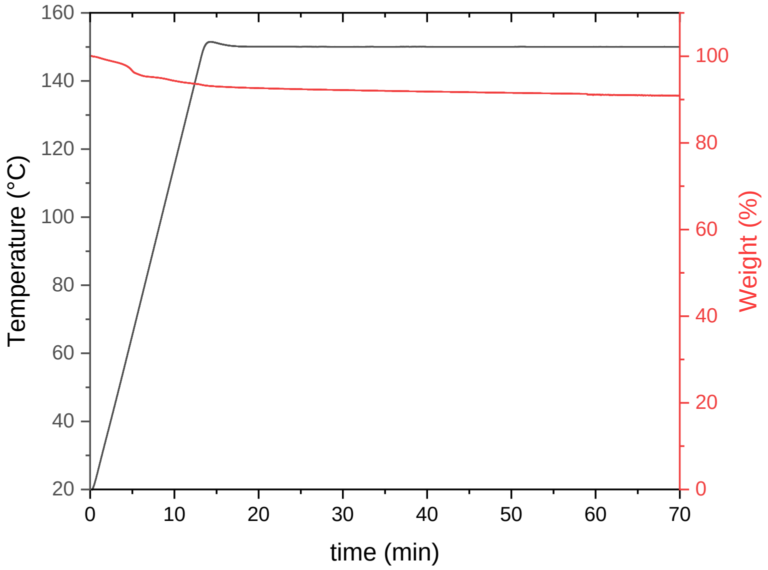

Figure S12. TGA measurement of the complex **1** at 150°C.

General procedure for the polymerization of lactide at room temperature

The polymerization was carried out under inert conditions. In a Braun Labmaster glovebox, a magnetically stirred reactor vessel (10 mL) was charged with lactide. The metal-complex and iPrOH (0.1 M solution of iPrOH in dichloromethane) were added in a 4mL vial and mixture was stirred for 5’ then added dichloromethane and transferred in the monomer. The reaction mixture was stirred at room temperature. During the reaction, small aliquots of the reaction mixture were sampled, dissolved in $CDCl_3$ and analyzed by ^{1}H NMR spectroscopy. At the end of the polymerization the product was precipitated in hexane then filtered and dried in a vacuum oven.

General procedure for the polymerization of lactide at high temperature

The polymerization was carried out under inert conditions. In a Braun Labmaster glovebox, a magnetically stirred reactor flask (50 mL) was charged with lactide. The metal-complex and 1 equivalent of BzOH (0.1 M solution of BzOH in toluene) were added in a 2 mL vial and the mixture was stirred for 5’ then transferred in the monomer and, finally, mixed to more equivalents of BzOH. The reaction mixture was stirred at 150°C. During the reaction, small aliquots of the reaction mixture were sampled, dissolved in $CDCl_3$ and analyzed by ^{1}H NMR spectroscopy. At the end of the polymerization the product was precipitated in hexane then filtered and dried in a vacuum oven.

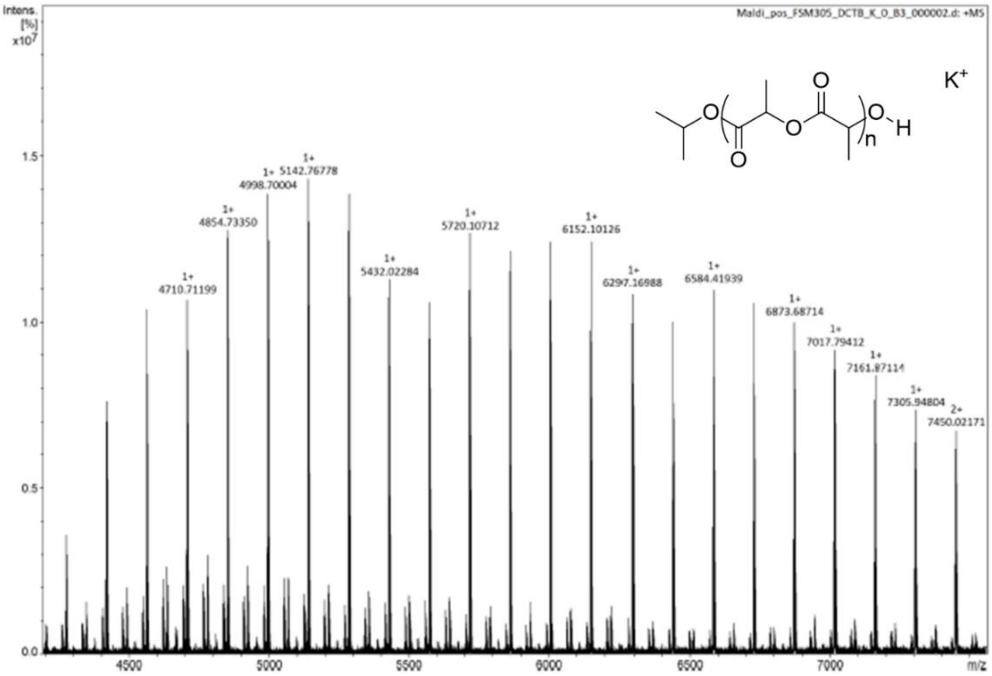

Figure S13. MALDI-TOF of P(rac-LA) sample run 2 Table 1.

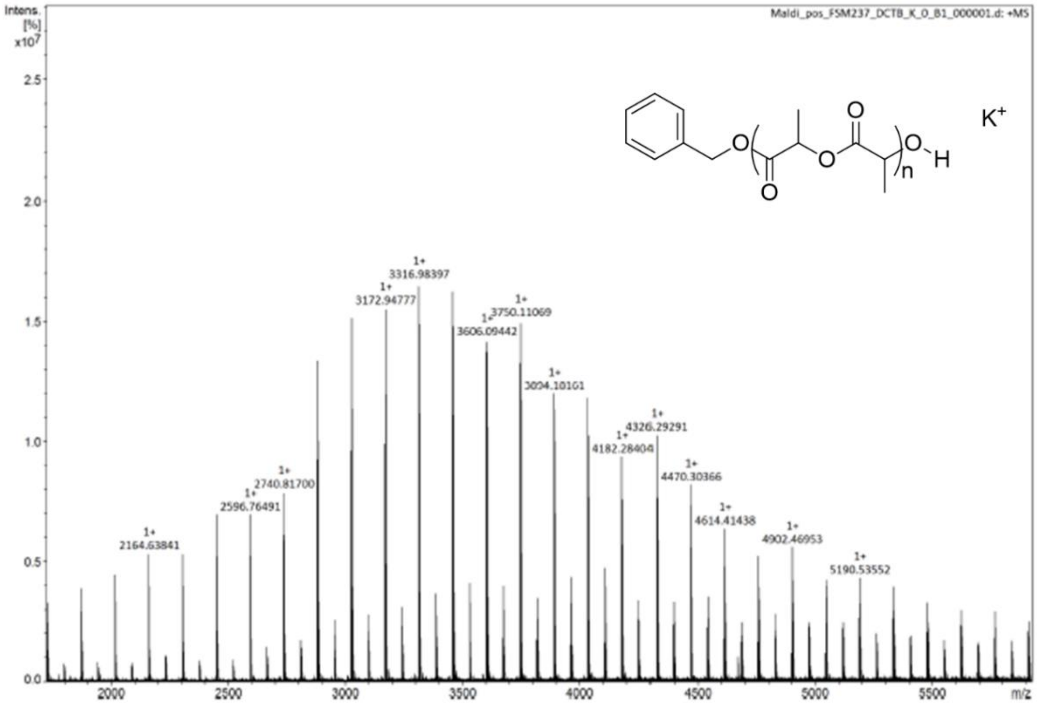

Figure S14. MALDI-TOF of P(L-LA) sample run 11 Table 1.

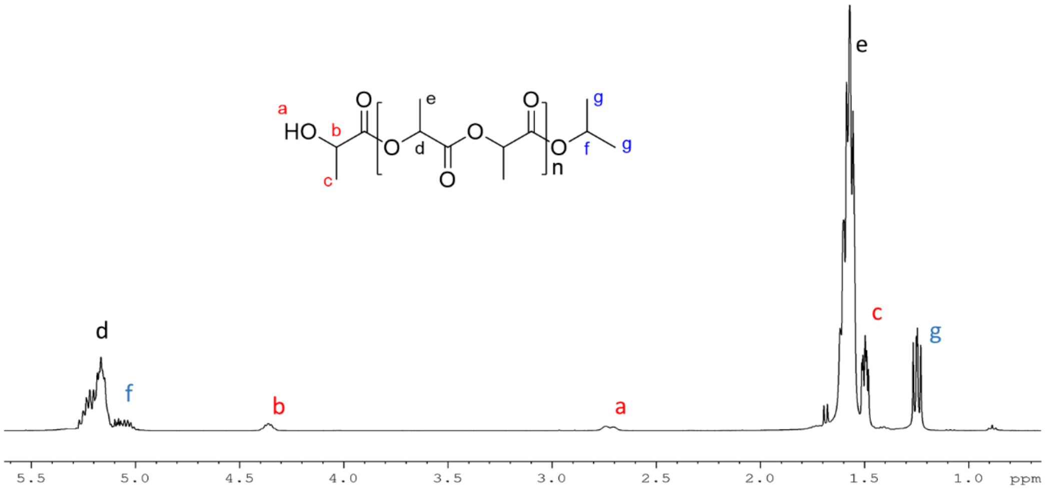

Figure S15. ^{1}H NMR spectrum (400 MHz, $CDCl_3$, 298 K) of a P(rac-LA) sample Table S1.

Istituto di Scienze e Tecnologie Chimiche "Giulio Natta" (SCITEC CNR)

Laboratorio di Caratterizzazione Molecolare

Tel: 02-23699.384 Fax: 02-23.62.946 e-mail: cmlab@ismac.cnr.it

Software: Empower Software

Report Method: GPCRel_Results

Sample Name: Losio_FSM222

Printed: 28/05/2021

Page: 1 of 2

INJECTION INFORMATION

Project Name: SEC_THF_2021

Sample Name: Losio_FSM222

Acq Method: Default600

Date Acquired: 27/05/2021 21.02.45

Channel: 410

Vial: 8

Sample Type: Broad Unknown

Processing Method: PS_Relativ_Mag21

Date Processed: 28/05/2021 9.58.17

Injection Volume: 150.00 ul

Injection: 1

Run Time: 60.00 Minutes

SYSTEM INFORMATION

System: W600+W717+PDA996+DRI410;THF Stab; 0.6;35°C; Deg

Columns: 3PL (Poli, Oligo, 50A 5 um)

SAMPLE DESCRIPTION: Losio PLA camp FSM222

Channel Description: 410+32 Baseline Noise: 0.054 Baseline Drift: 3.857

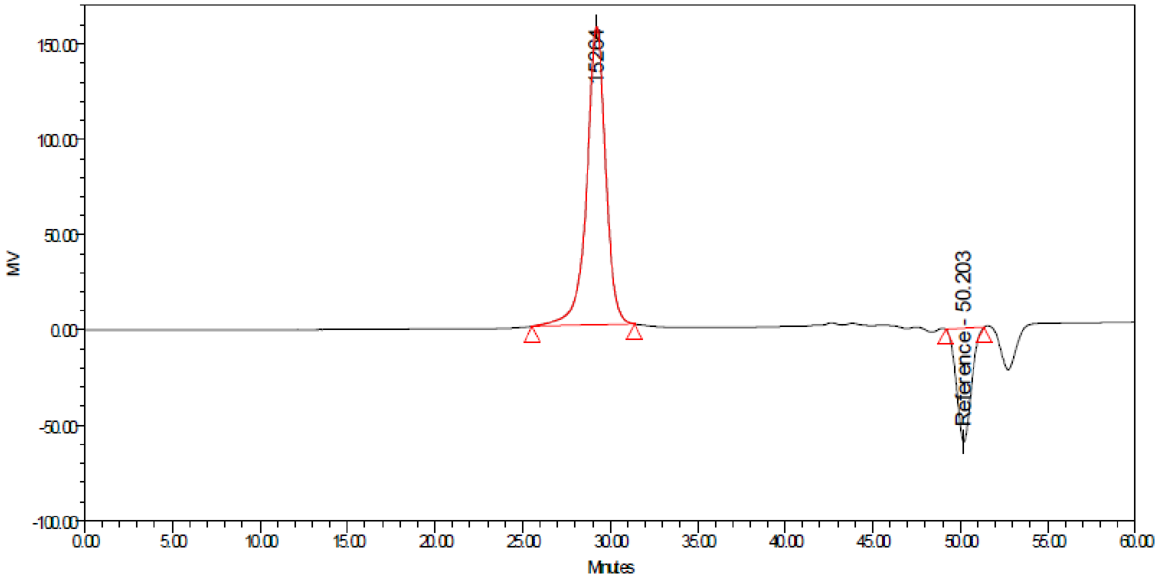

Peak Results

	RT	Area	%Area	Height	%Height	Int Type	Signal Noise
1	29.226	11755690	78.07	156475	72.38	bb	2885.942
2	50.203	3301748	21.93	-59716	27.62	bb	1101.364

GPC Relative Calibration Results

	Ret Time	Mp	Mn	Mw	Mz	Mw/Mn	Mz/Mw
1	29.226	15264	15046	17857	25757	1.19	1.44
2	50.203						

Figure S16. GPC analysis of sample 12 Table 1.

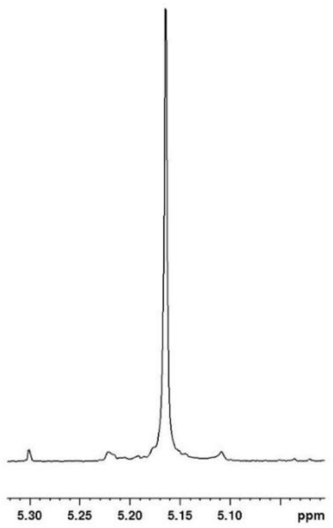

Figure S17. Methine region of the homonuclear decoupled ^{1}H NMR spectrum ($CDCl_3$, RT, 400MHz) of a P(L-LA) sample run 12 Table 1.

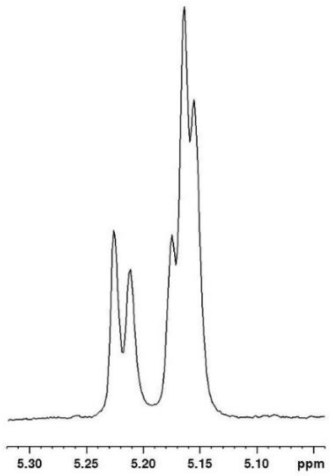

Figure S18. Methine region of the homonuclear decoupled ^{1}H NMR spectrum ($CDCl_3$, RT, 400MHz) of a P(D,L-LA) sample run 2 Table 1.

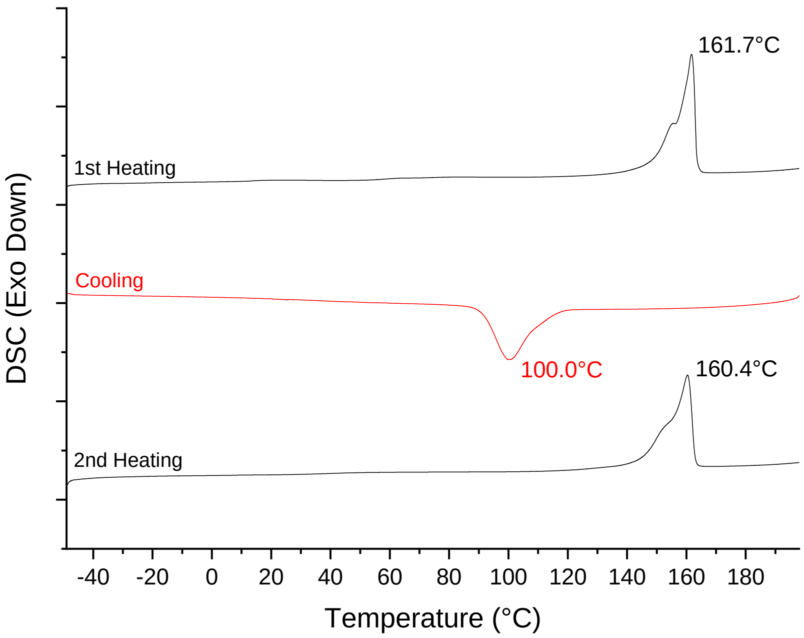

Figure S19. DSC measurement of a P(L-LA) sample run 10 Table 1.

Table S1. Polymerization of lactides promoted by 1.[a]

Run	Mon	[LA]/[Zn]/[ROH]	T (°C)	Time (min)	Conv (%)	TOF (h^{-1})
**1**	rac-LA	100/1/0	25	8	61	457
**2**	rac-LA	100/1/1	25	0.5	22	2640
**3**	rac-LA	400/1/1	25	0.5	59	28320
**4**	L-LA	400/1/1	25	0.5	54	25920
**5**	L-LA	400/1/40	25	0.5	31	14880
**6**	L-LA	400/1/100	25	2	67	8040
**7** [d]	L-LA	1000/1/2	25	2	56	16800
**8**[e]	L-LA	1000/1/2	25	2	68	20400
**9**[f]	L-LA	1000/1/0	150	4	23	3450
**10** [f]	L-LA	1000/1/10	150	1	100	60000
**11** [f]	L-LA	1000/1/50	150	0.5	100	120000
**12** [f]	L-LA	5000/1/50	150	5	82	49200
**13**[e]	L-LA	400/1/1	25	0.5	0.5	12000

[a]All reactions were carried out by using 10 µmol of Zn catalyst at 25 °C in CH_2Cl_2 (2 mL), ROH was ^{i}PrOH for runs at 25 °C and BzOH for runs at 150 °C. [b]Experimental M_n (corrected using factor of 0.58) and *Đ* values were determined by GPC analysis in THF using polystyrene standards. [c]Calculated M^{th}(KDa) =144.14 x ([LA]/[ROH]) x conversion of LA.[d]LA purified by a single crystallization [e] unpurified LA. [f]technical grade purity LA, solvent free. [e] Reaction performed in THF.

General procedure for the depolymerization of polylactide in solution

The depolymerization reaction was carried out under an inert atmosphere. In a Braun Labmaster glovebox, a magnetically stirred reactor vessel (10 mL) was charged with polylactide and MeOH. In a vial (4mL) the metal-complex was dissolved in THF and added to the polymer. The reaction mixture was stirred at room temperature. At desired times, small aliquots of the reaction mixture were sampled, dissolved in $CDCl_3$ or CD_2Cl_2 and analyzed by 1H NMR spectroscopy. At the end of the depolymerization the reaction was stopped with CH_2Cl_2 and dried under vacuum.

PLA sample	Mn (KDa)	Đ	T_f (°C)
P(L-LA) 1	160	1.2	179
P(D,L-LA)	13	1.1	
P(L-LA)2	30	1.1	171
P(L-LA)3	48	1.8	170
P(L-LA)4	92	1.5	178
P(L-LA) cup	58	1.9	150
P(L-LA)filament	20	2.9	168

Table S2. Number average molecular weight (Mn), dispersity (Đ) and Tf of PLA samples used for depolymerization reactions.

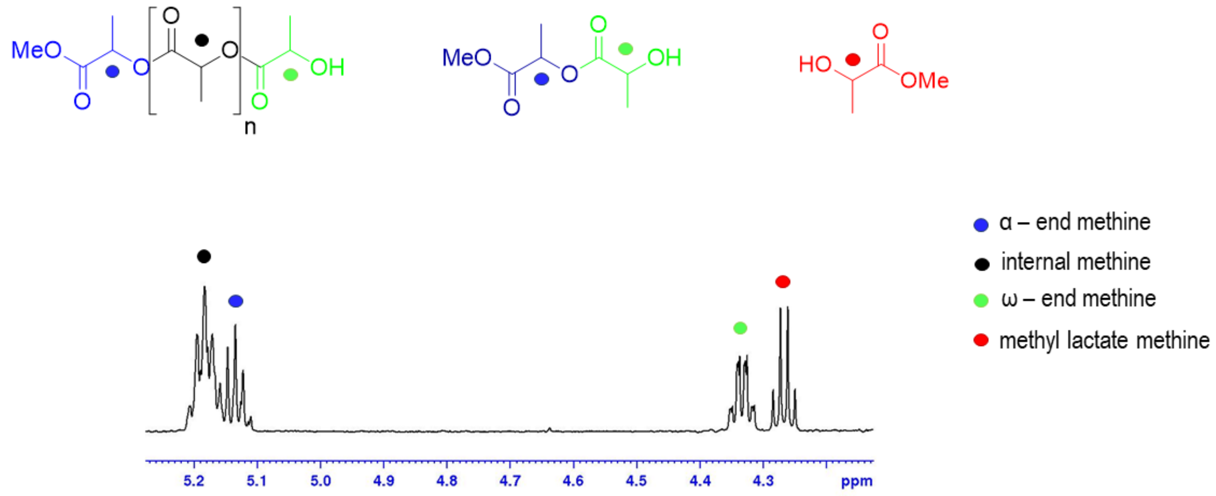

Figure S20. ^{1}H NMR spectrum (600 MHz, CD_2Cl_2, 298 K) depolymerized PLLA.

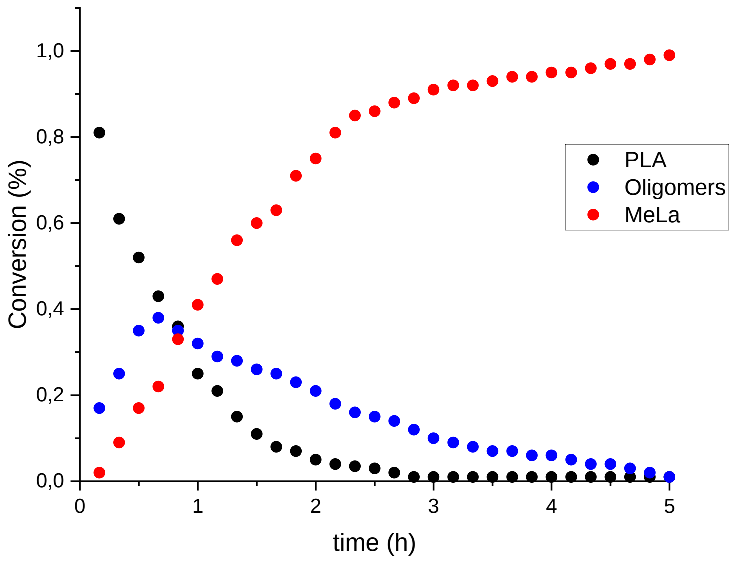

Figure S21. Kinetic profile for degradation of a poly (rac-LA) sample ([PLA]0 = 0.44 M; [polyester linkages]0/[Zn]0 = 60; CH_2Cl_2 (0.45 mL) as solvent; MeOH 0.05mL).

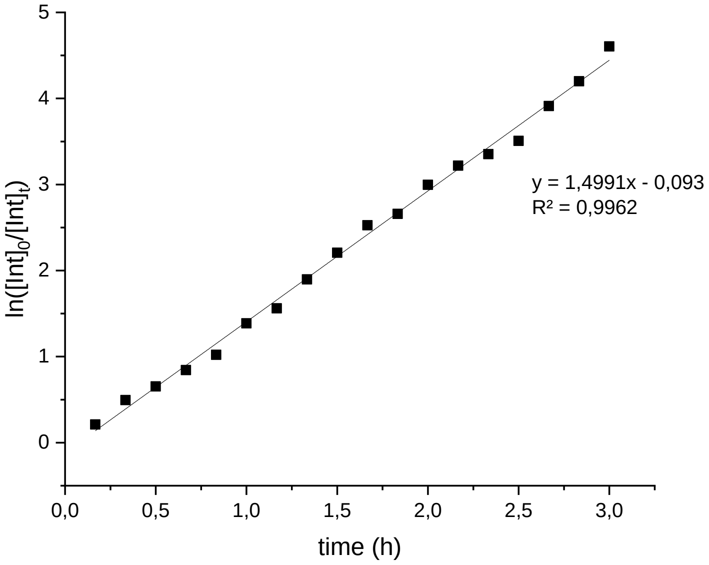

Figure S22. Pseudofirst-order kinetic plot for degradation of poly(rac) lactide ([PLA]0 = 0.44 M; [polyester linkages]0/[Zn]0 = 60; CH_2Cl_2 (0.45 mL) as solvent; MeOH 0.05mL). Pseudofirst-order rate is = 1.50 h^{-1} at 25°C (R^2 = 0.9962).

General procedure for the depolymerization of polylactide solvent free

The depolymerization reaction was carried out under an inert atmosphere. In a Braun Labmaster glovebox, a magnetically stirred reactor vessel (10 mL) was charged with polylactide. In a vial (4mL) the metal-complex was dissolved in MeOH or EtOH and added to the polymer. The reaction mixture was stirred at room temperature. At desired times, small aliquots of the reaction mixture were sampled, dissolved in $CDCl_3$ or C_6D_6 and analyzed by ^1H NMR spectroscopy. At the end of the depolymerization the reaction was stopped with CH_2Cl_2 and dried under vacuum.

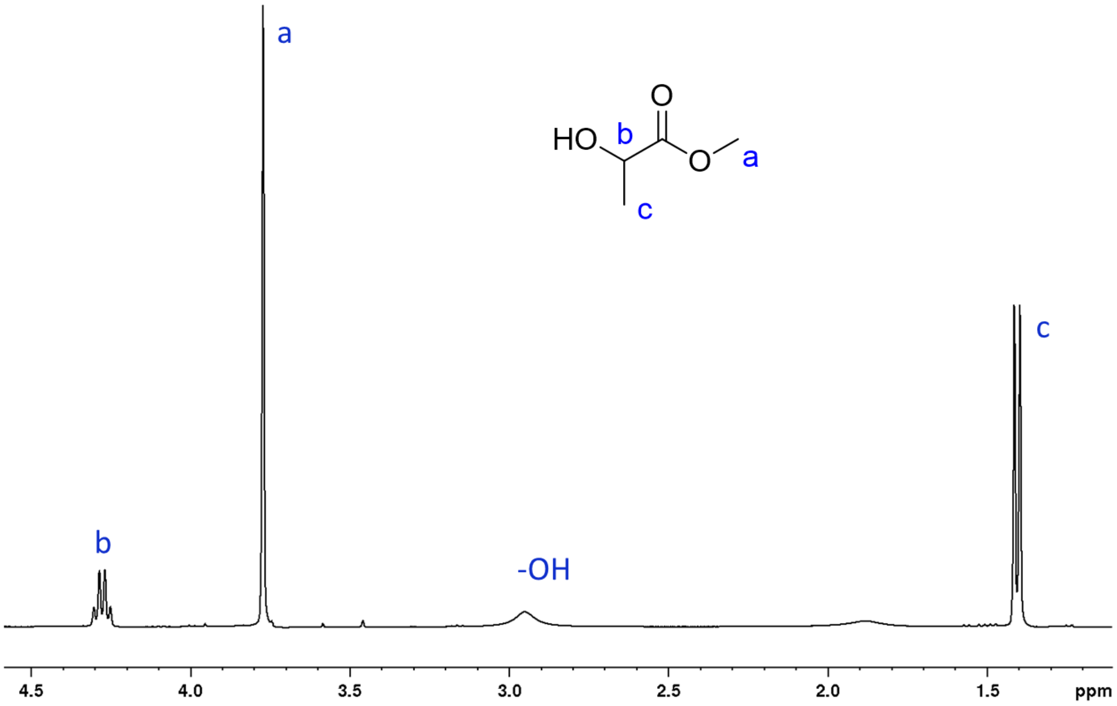

Figure S23. ^{1}H NMR spectrum (400 MHz, $CDCl_3$, 298 K) of methyl-lactide (Me-La).

^{1}H NMR (400 MHz, $CDCl_3$, 298 K): δ 4.27 (q, J = 6.7Hz, 1H, H_b), 3.77 (s, 3H, H_a), 2.95 (br, 1H, -OH), 1.40 (d, J = 6.8Hz, 3H, H_c).

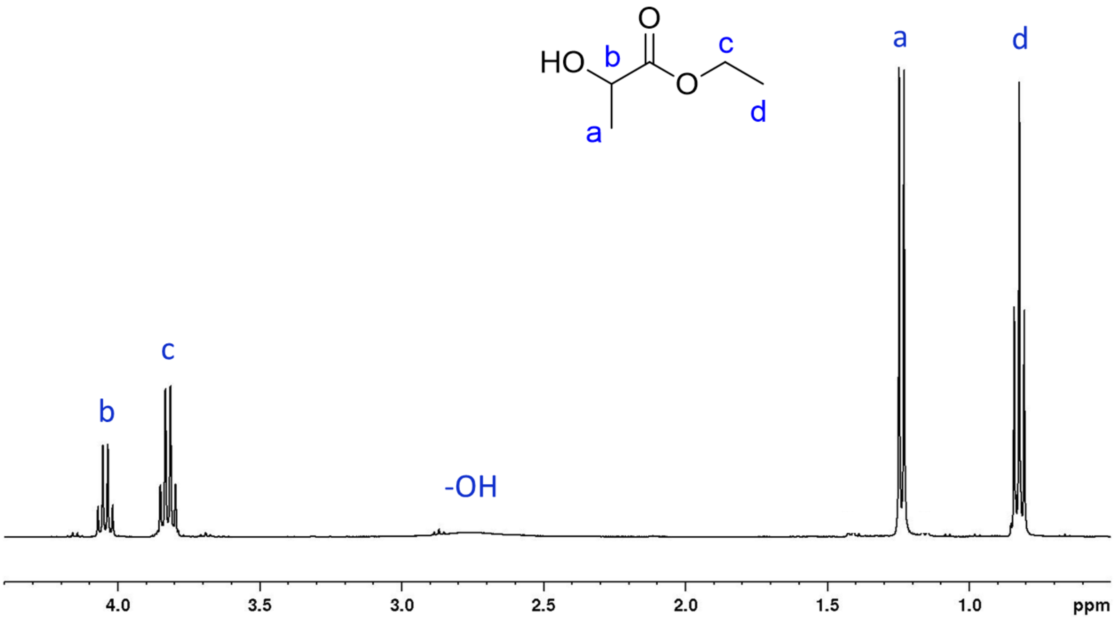

Figure S24. ^{1}H NMR spectrum (400 MHz, C_6D_6, 298 K) of ethyl-lactide (Et-La).

^{1}H NMR (400 MHz, C_6D_6, 298 K): δ 4.04 (q, J = 6.9Hz, 1H, H_b), 3.82 (q, J = 7.2Hz, 2H, H_c), 2.76 (br, 1H, -OH), 1.23 (d, J = 6.9Hz, 3H, H_a), 0.82 (t, J = 7.1Hz, 3H, H_d).

Table S3. Methanolysis of PLAs performed in THF solution.[a]

Run	Sample	Mn[b] (KDa)	Time (h)	[d]XInt	[c]Sme-La	[c]YMe-La
**1**	P(L-LA)	30	1	100	61	61
			1.5	100	100	100
**2**	P(L-LA)	48	1	100	74	74
			1.5	100	100	100
**3**	P(L-LA)	92	1	100	77	77
			1.5	100	100	100
**4**	P(L-LA)	14	0.5	94	74	70
			1	97	95	92
**5**	P(D,L-LA)	13	0.5	55	27	15
			1	55	48	27
**6**	P(D,L-LA)	44	1	75	69	52
			2	88	89	78
**7** [d]	P(L-LA)	30	2	89	38	34
			4.5	97	61	60
			24	100	100	100
**8** [d]	PLA cup	58	2	100	47	47
			4.5	100	100	100
**9** [d]	PLA filament	20	2	84	26	22
			4.5	100	62	62

[a]All reactions were carried out by using 5 µmol of Zn catalyst (0.6 mol % relative to ester linkages) in 1.8 mL of THF, with 0.2 mL of MeOH [b] M_n = the experimental (GPC) values of the recorded molecular weight of the polymer. [c]Determinated by ^{1}H NMR; [d]reaction performed in air.
